# Sirtuins, brain and cognition: A review of resveratrol effects

**DOI:** 10.1016/j.ibror.2020.06.004

**Published:** 2020-06-26

**Authors:** Daniel Silva Moraes, Daniele Cristina Moreira, João Marcus Oliveira Andrade, Sérgio Henrique Sousa Santos

**Affiliations:** a Postgraduate Program in Health Science, Universidade Estadual de Montes Claros (Unimontes), Montes Claros, Minas Gerais, Brazil; b Institute of Agricultural Sciences (ICA), Food Engineering, Universidade Federal de Minas Gerais, Montes Claros, Minas Gerais, Brazil

**Keywords:** Sirt, Polyphenol, Phytochemistry family, Wine, Central nervous system

## Abstract

Sirtuins (SIRTs) are a protein family with high preservation degree among evolutionary scale. SIRTs are histone deacetylases regulatory enzymes of genetic material deeply involved in numerous physiological tasks including metabolism, brain function and aging. Mammals sirtuins comprise seven enzymatic components (SIRT1–SIRT7). The highest studied sirtuin is SIRT1, which plays an essential position in the prevention and evolution of neuro-disorders. Resveratrol (3,5,4-trihydroxystylbene) (RSV) is a polyphenol, which belongs to a family compounds identified as stilbenes, predominantly concentrated in grapes and red wine. RSV is the must studied Sirtuin activator and is used as food supplementary compound. Resveratrol exhibits strong antioxidant activity, reducing free radicals, diminishing quinone-reductase-2 activity and exerting positive regulation of several endogenous enzymes. Resveratrol is also able to inhibit pro-inflammatory factors, reducing the stimulation of the nuclear factor kB (NF-kB) and the release of endogenous cytokines. Resveratrol treatment can modulate multiple signaling pathway effectors related to programmed cell death, cell survival, and synaptic plasticity. In this context, the present review looks over news and the role of Sirtuins activation and resveratrol effects on modulating target genes, cognition and neurodegenerative disorders.

## Introduction

Sirtuins are enzymes catalogued as histone deacetylases (HDACs), which are proteins able to inhibit gene transcription for its skill to delete acetyl portions of the ε-acetamido unit in lysine inside histones (de Ruijter et al., 2003). The amino portion of preserved lysine contained in histone extremities is able to be reversible acetylated and deacetylated, which plays a relevant position on gene expression (Li et al., 2007). The histone modifications also play a role modulating DNA damage, genetic instability, pro-inflammatory genes and premature aging (Krishnan et al., 2011; Ogiwara et al., 2011; Sarg et al., 2002; Vempati et al., 2010; Yuan et al., 2009). This progression can modify the chromatin arrangement between euchromatin and heterochromatin, in order to activate DNA restoration process to impaired locations (Yao and Rahman, 2012).

NF-κB-dependent pro-inflammatory genes transcript may also be regulated by HDACs through deacetylation of non-histone proteins (de Ruijter et al., 2003; Yao and Rahman, 2012). To date, several isoforms of HDAC have been recognized and grouped into classes (de Ruijter et al., 2003). Among these classes, the Sirtuins can be highlight as Class III members that use NAD + as a cofactor (Alcendor et al., 2007; Finkel et al., 2009; Lavu et al., 2008; Rajendrasozhan et al., 2009). The first sirtuin family protein was identified in *Saccharomyces Cerevisiae* (Rine and Herskowitz, 1987), being appointed as regulator of silent information 2 (Sir2) gene. Sir2, which is also expressed in *Caenorhabditis Elegans* and *Drosophila melanogaster,* was associated to aging and longevity among other functions. Considering the mammals, Sirtuin 1 (SIRT1) was the first identified. Subsequently, other sirtuin family genes emerged constituting a total of seven (SIRT1 to SIRT7) (Donmez, 2012; Kelly, 2010a, b).

SIRTs are regulatory genes that modulate a high diversity of epigenetic factors. These proteins play a primary function in the body's reactions to diverse stress forms and toxicity. Sirtuins adjust animal’s lifetime interfering with biological factors related to metabolic alterations and also aging in mammals (Michan and Sinclair, 2007; Paraiso et al., 2013). The seven mammals’ sirtuins (SIRT1 to SIRT7) have being extensively studied. Previous researches have demonstrated changes at the cellular level locations for each Sirtuin. The SIRT3, SIRT4 and SIRT5 are localized in mitochondria, while SIRT6 and SIRT7 are mainly nuclear, while SIRT2 and SIRT1 are both present in the nuclei and in the cytosol. (Paraiso et al., 2013; Lin et al., 2013). It is worth to repeat that a key function of nuclear sirtuins (SIRT 1, 2, 6 and 7) is the gene inflammation regulatory task.

Emerging as a promising tool on regulating SIRTs, resveratrol (RSV) is a polyphenol with several beneficial properties through their antioxidant and anti-inflammatory effects, modulating several cascades and effectors involved in the brain and cognitive regulation, specially SIRT1-mediated (Andrade et al., 2019; Sarubbo et al., 2018a, b).

## Sirtuins and brain

Several studies demonstrated that sirtuins plays a crucial role on aging, neural disorders and metabolic syndrome (Brachmann et al., 1995; Smith et al., 2000). Considering the 7 mammalian sirtuins, the overexpression of SIRT1 and/or its stimulation by certain natural chemical structures (resveratrol for instance), enhance health and life span (Chandrasekaran et al., 2019). SIRT1 modulates several gene components, however there are some priority transcription factors. We are able to highlight among these transcription factors the p53 (Vaziri et al., 2001), FoxO family members (Brunet et al., 2004), NF-κB (Yeung et al., 2004) and PGC-1α (Dominy et al., 2010; Rodgers et al., 2008). Changes in these components due to their deacetylation, alters the cell's life cycle and also the energy metabolism. SIRT1 cleaves NAD + into nicotinamide and 10-O-acetyl-ADP-ribose (Tanner et al., 2000) or 20- and 30-O-acetyl-ADP-ribose (Jackson et al., 2003) and therefore deacetylate lysine residues. SIRT1 activities require and increase NAD + cell content, which means a reduction in cell energy stock (Chalkiadaki and Guarente, 2012). SIRT1 activation by resveratrol is able to protect mice against high-fat induced obesity and insulin disturbance (Canto et al., 2012; Lagouge et al., 2006; Rajman et al., 2018). The described activation produces a decline in PGC-1α /acetylation and an increased activity of the same protein (Rodgers et al., 2008; Gerhart-Hines et al., 2007; Nemoto et al., 2005).

Recent findings showed the SIRT1 ability to improve mitochondrial breathing. The NAD + consumption pathways maintain cellular homeostasis protecting the dorsal root ganglia neurons from peripherals damage induced by high-fat diet (HFD), thus preventing neuropathy. It is important to note that the overexpression of SIRT1 was capable to avoid and treat the peripheral neuropathy stimulated by HFD. The authors suggested a mitophagia associated with NEDD4 that improves mitochondrial breathing capacity, increasing axonal development and reparation. SIRT1 is essential to this route by regulating mitochondrial role in the marginal nerve throughout PGC1-α modulation (Chandrasekaran et al., 2019). Cell culture researches showed that SIRT1 is detect in the nucleus of several cell lines (Michan and Sinclair, 2007) producing deacetylation activity of several transcription factors. SIRT1 protein, nevertheless, seems to have nuclear signs exportation and modulates the transport between the cytoplasm and the nucleus (Tanner et al., 2000; Sugino et al., 2010). Extranuclear location, particularly in mitochondria, has also been observed (Aquilano et al., 2013). Immunohistochemistry analyzes of samples with high SIRT1 expression demonstrated a clear location in the nuclei of CA1 neurons in the hippocampus.

The adverse effects of cadmium chloride in the maintenance and spatial-memory tasks in rats confirm the participation of reactive oxygen species, reduction of intracellular glutathione amount, and activation of inherent cell-death in this course. Cadmium chloride produces in the mice hippocampus a continued stimulation of Endoplasmic Reticulum (ER) with parallel decrease in the SIRT1 level and lower activity of the SIRT1/AMPK/Akt axis. Confirming this data, the rats treated with cadmium chloride and resveratrol presented improved memories and reduced reactive oxygen species generation with improved GSH and increased levels of Bcl-2 mediated by negative regulation of GAAD-153 (CHOP), in a mechanism dependent on SIRT1/AMPK/Akt. In complement, resveratrol inhibited cadmium chloride-induced hippocampal apoptosis, avoiding ER stress and subsequent initiation of proapoptotic genes downstream (Shati, 2019).

Nuclear factor of activated B-cells (NF-κB) is involved in physiological inflammatory processes and thus representing a promising target for inflammation-based neuronal therapy. Yang et al. demonstrated that resveratrol reduced inflammatory damage and promoted microglia polarization to the M2 phenotype in LPS-induced neuroinflammation. In addition, resveratrol ameliorated LPS-induced sickness behavior in mice. The promoting effects of resveratrol on M2 polarization were attenuated by knocking down PGC-1α. PGC-1α not only suppressed LPS-evoked M1 marker expression by inhibition of NF-κB activity but also increased M2 marker expression by coactivation of the STAT6 and STAT3 pathways (Yang et al., 2017). In other study, RSV inhibited the activation of NLRP3 and NF-κB in the hippocampal region caused by deficiency of estrogen, ameliorating ovariectomy-induced anxiety and depression-like behaviors (Liu et al., 2019). Fan et al. showed that SIRT1 mediates the anxiolytic effect of apelin-13 in chronic normobaric hypoxia-treated mice through the inhibition of NF-κB pathway. These results imply that dysfunction of the apelin-SIRT1-NF-κB axis in hippocampus represents a potential mechanism that results in the induction of neuroinflammation and reduction in neuroprotection, thus induces anxiety-like behavior in chronic normobaric hypoxia-treated mice (Fan et al., 2018). Altogether, these studies indicate the important role of the NF-κB inhibition in the resveratrol’s neuroprotective effect.

## Resveratrol, brain and cognitive function

Resveratrol (3,4′,5-trihydroxystilbene; C14H12O3) (RSV) is a polyphenolic phytoalexin found in grapes, berries, peanuts, and wines, and belongs to a family of polyphenolic compounds known as stilbenes. RSV has been viewed as an antioxidant, anti-inflammatory, anti-apoptotic, anti-obesity and anticancer agent (Cvejic et al., 2010; Sahebkar et al., 2015; Shi et al., 2014). RSV is a low molecular weight compound with antimicrobial activity. There are two RSV forms, the -trans and -cis isomers. RSV plays a central role in the famous “French Paradox”, that showed the inverse correlation between the occurrence of cardiovascular disease and the intake of red wine in French population. Today, RSV has been viewed as a neuroprotective agent.

Investigational data suggest that resveratrol (RSV) induces antinociception in the nervous system periphery through the opioid activation of receptors and by the release of endogenous and endocannabinoid opioids. RSV induces the antinociceptive effect against the inflammatory carrageenan agent. Two theories have been proposed to explain the effects of peripheral RSV antinociceptive involvement: (i) endocannabinoid anandamide (AEA) and 2-AG releasing subsequent stimulation subsequent to CB1R receptor activity and opioid receptor (OR) associated with an opioid endogen; (ii) opioids release the stimulation subsequently caused by OR activation and cause indirect activation of CB1R with the AEA used (Oliveira et al., 2019).

## Resveratrol and cognition

Postoperative cognitive diseases represent an important neurological problem in almost 25 % of the elderly people. In fact, this cognitive dysfunction induces hippocampus overproduction of proinflammatory molecules [i.e. tumor necrosis fator alpha (TNF-α) and interleukin (IL) -1B]. Isoflurane anesthesia damages synaptic plasticity leading to neurological problems followed by cognitive dysfunction (Rachal Pugh et al., 2001; Terrando et al., 2010). In other study, elderly mice treated with intraperitoneal resveratrol 100 mg/kg in a total of 7 days, attenuating isoflurane hippocampal-dependent damage through anti-inflammatory effects (Toth et al., 2014). Considering the possible molecular routes mechanisms modulating these effects, SIRT1 involvement raised a great interest (Hasegawa and Yoshikawa, 2008). In particular, neuronal SIRT1 deacetylate p53 in the Lys residues protecting multiple cells against apoptosis induced by DNA damage (Du et al., 2014) Fig. 1. Confirming these data, cell primary neurons studies in mouse and rat models showed that SIRT1 intermediate neuronal protection working as a central actor in combating neurodegenerative disorders (Chen et al., 2015).

**Fig. 1:**
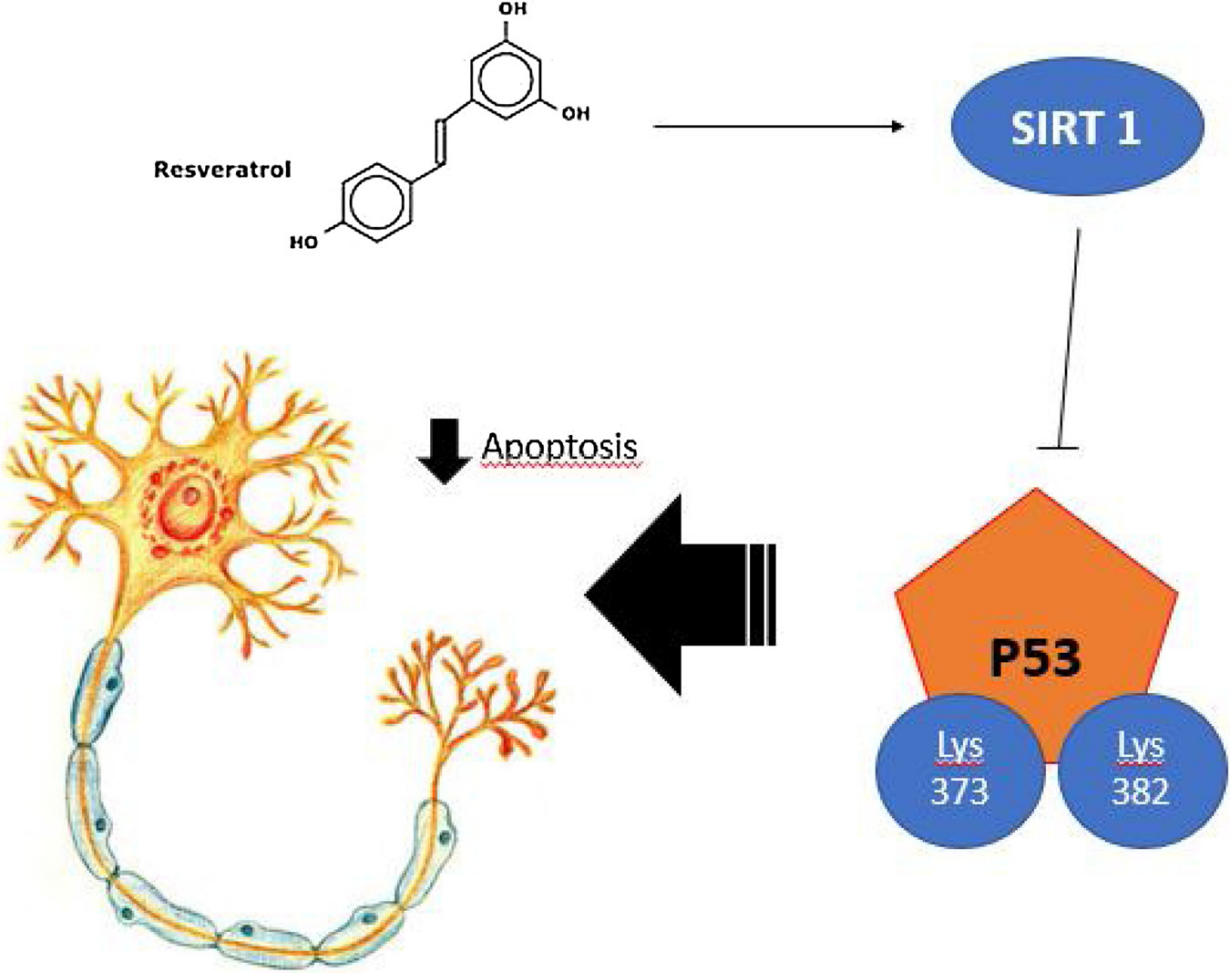
Resveratrol improves cognition through an anti-oxidative mechanism by SIRT1-mediated deacetylation. Unpaired electrons escape the mitochondrial electron transport chain and react with molecular oxygen to produce superoxide, resulting in oxidative stress. Resveratrol acts on SIRT1, which induces ROS detoxifying enzymes that eliminate superoxide.

Resveratrol plays a central role as the key polyphenol capable to modulate SIRT1 expression and its main effects. SIRT1 activation by RSV can produce neural malleability in the hippocampus area (Hasegawa and Yoshikawa, 2008). This path relevance is also confirmed in a mouse neuropathology induced model similar to Alzheimer's disease (AD). The authors showed that SIRT1 activation by RSV (30 mg/kg/day for 8 weeks) reduced Tau protein phosphorylation induced by the brain streptozotocin injection. These data confirms resveratrol role defending the hippocampal neuronal area of Tau and memories hyperphosphorylation commitment (Du et al., 2014). A recent study showed that RSV significantly increased SIRT1 expression inhibiting the memory impairment. The results were associated with increased acetylcholinesterase, malondialdehyde and reduced superoxide dismutase (SOD) and glutathione levels in a diabetic rat model with concomitant Alzheimer's disease (Ma et al., 2019).

RSV also plays a role on improving the activation of AMPK-protein kinase (AMPK), which causes neurogenesis and mitochondrial biogenesis, thus stimulating the biogenesis of neural differentiation in neurons. These properties are SIRT1 independent, considering the results obtained using SIRT1 inhibitors or studies performed in the SIRT1 knockout mice brain (Dasgupta and Milbrandt, 2007). It is now clear the existence of a close interaction between SIRT1 and AMPK (Canto et al., 2009). An Alzheimer disease mice model proved that exist a balance concerning SIRT1 and AMPK signaling linked to inflammatory changes which are required for the RSV protective effects against Ab formation and cognitive plaque loss (Porquet et al., 2014). The confirmation of a neural RSV effect was further validated in H19-7 rat neuronal hippocampus cells in vitro, where a 2 -h pre-treatment with RSV (75 mM) diminished the oxidative damage produced by Ab and reducing crucial synaptic proteins development and malleability (Rege et al., 2015). Other impaired source in AD, which can be redeemed by RSV, it is the neurovascular-coupling. An aging mice-model with cerebrovascular deficits was rescued by RSV treatment improving cortical neurovascular-coupling responses. The main effects were intermediated by downregulation of cortical NADPH production and ROS derived reduced effects (Toth et al., 2014) Fig. 2. Considering that SIRT1 constrains NADPH oxidase outcomes in rat aorta and defends against endothelial dysfunction, this path is fundamental to understand the bond between RSV and cerebromicrovascular vessel endothelial-function (Zarzuelo et al., 2013).

**Fig. 2:**
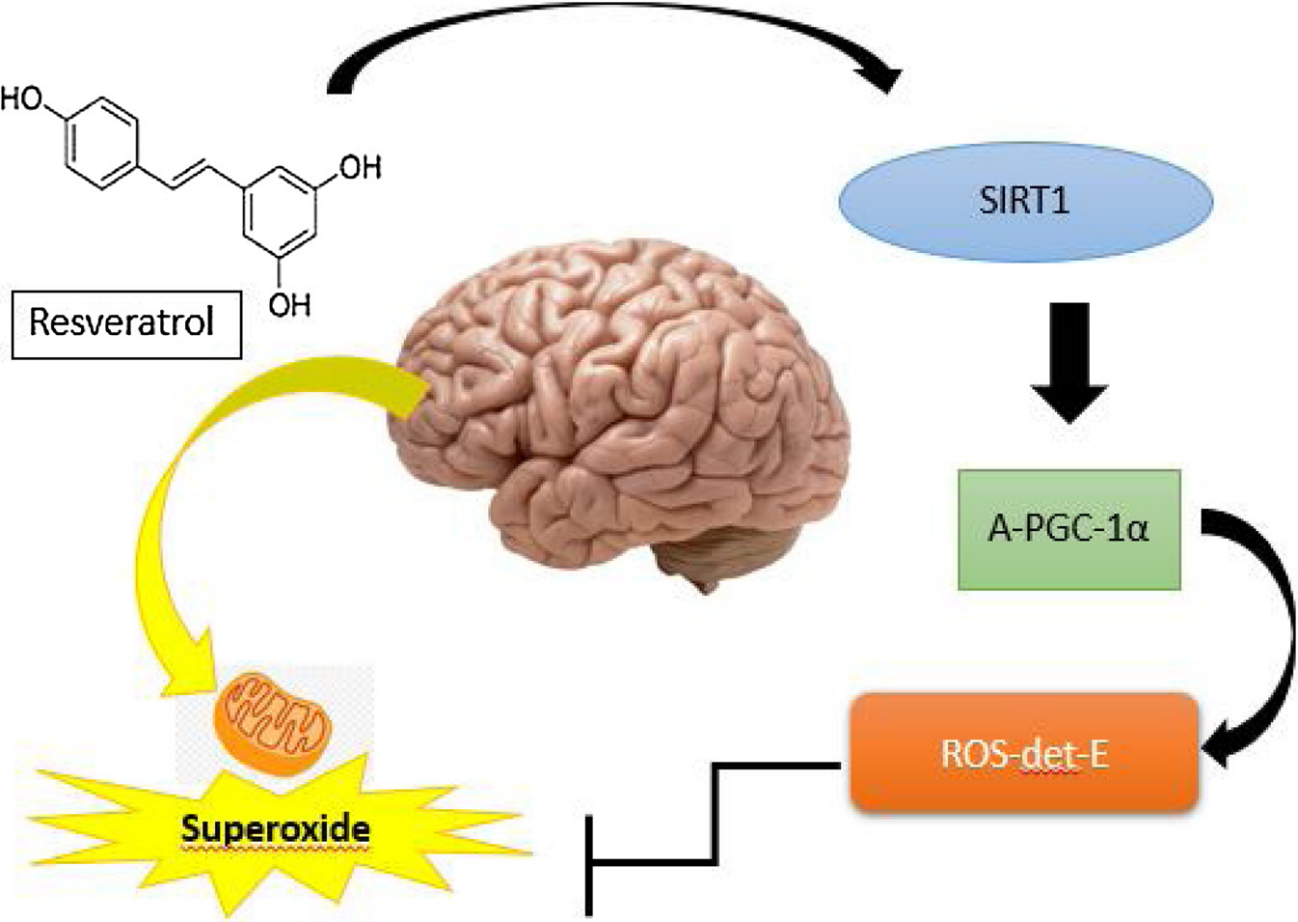
SIRT1 is capable of deacetylate p53 in the Lys residues (Lys373 and / or Lys382) and protect multiple cells against Apoptosis induced by DNA damage.

The neurogenic effect of resveratrol is also an important protective factor for the central nervous system. RSV is able to cross the brain barrier through the circulatory system and causes improvement of antioxidant enzymes, in addition it extends the effects of the pathways linked to SIRT1 and induces glial activation, helping to increase neurogenesis in the hippocampus. Other results are the decrease in the expression of the amyloid precursor protein and the improvement of the special working memory (Gomes et al., 2018).

In a complementary way to SIRT1/AMPK involvement on the brain function, an analysis of gene expression across the genome showed as that resveratrol balance the hippocampal gene expression involved in neuronal origin and synaptic malleability (Hdac4, Hat1, Wnt7a, ApoE) and reduction of Jak-Stat pro-inflammatory signaling (IL-15, IL-22, Socs2 and Socs5) in streptozotocin-induced diabetic rats (Thomas et al., 2014). The use of resveratrol led to the expression of the hippocampal nerve growing component, decreasing pyramidal cell mortality in a hippocampus CA1 region, increasing spatial working memory in a vascular dementia rat-model (Anastacio et al., 2014). More indications on the neural effect of RSV on the vascular associated dementia derived from a study showing how a permanent bilateral carotid (vessel) occlusion in a rat model, can be treated with daily RSV administration, improving memory/learning as assessed by the Morris water maze test. The exhaust dormancy and escape distances were expressively lower in RSV animals. In addition, after resveratrol, malonyldialdehyde amount, an oxidation indicator stress in neuronal disorders, diminished in the cortex and hippocampus; inversely, RSV treatment produced an increase in superoxide dismutase effects and glutathione levels (Ma et al., 2013).

In recent studies about resveratrol and brain, Zoe et al. showed that RSV may attenuate the inflammatory response and relieve traumatic brain injury by reducing reactive oxygen species production and inhibiting NLR family pyrin domain containing 3 (NLRP3) activation. The effect of resveratrol on NLRP3 inflammasome and reactive oxygen species may also be SIRT1 dependent (Zou et al., 2018). In other study, RSV increased the expression of genes encoding known antioxidants and anti-aging factors (SIRT1 and SIRT3) in Alzheimer's disease patients (Cosin-Tomas et al., 2019). Le et al. demonstrated that resveratrol plays a neuroprotective role in neonatal hypoxic-ischemic brain injury by activating SIRT1 to inhibit HMGB1/TLR4/MyD88/NF-kB signalling and subsequent neuroinflammatory responses (Le et al., 2019). Additionally, Shen et al. showed that the neuroprotective effect of RSV on chronic unpredictable mild stress -induced cognitive impairment may rely on activating SIRT1/miR-134 pathway and then upregulating its downstream element-binding protein (CREB) and brain derived neurotrophic factor (BDNF) expression in hippocampus (Shen et al., 2018). Finally, RSV protected against learning and memory impairments in juvenile animals fed with a high-caloric diet, possibly via upregulation of p16 or downregulation of PPAR in the hippocampal CA1 region (Xu et al., 2018).

## Human treatment

Aging-related dementia is globally increasing substantially, parallel to the "grayish" world population (Hirtz et al., 2007). Importantly, recent global data indicate that mild cognitive problems disturbs 5.5–7.7% of individuals over 60 years old and 22 % of people over 70 (Apostolo et al., 2016), most often in those with neuropsychiatric symptoms (Bidzan et al., 2017). Considering this epidemiological data, it is essential to explore new tools that can downgrade dementia advance. The decrease in cognitive capacity and dementia in adults has been investigated and several of its possible causes have been pointed out, among these genetic, nutritional and metabolic factors (Flirski and Sobow, 2005; Lahiri et al., 2007). Vascular injuries and inflammatory factors have been pointed as possible causes for these complications of the central nervous system (Jiang et al., 2017; Zhu et al., 2004). The evidences suggests that RSV, with all its effects cited throughout this review, may be a good option with neuroprotective actions and could have positive effects against the deterioration of human cognition. Some vegetables also seem to inhibit the evolution of neuronal problems (Cicero et al., 2018). Indeed, in addition to its helpful properties on the central nervous system, RSV appears to be capable to actuate on numerous cellular mechanisms/signaling and consequently produce a diversity of biological results, theoretically valuable to elderly diseases, (evidently confirmed in randomized clinical-trials) (Erdogan and Vang, 2016). In particularly, despite RSV presented controversial data on the lipid profile (Cicero et al., 2017), this polyphenol also appears to be effective on treating several Metabolic Syndrome (MS) constituents, such as overweight, insulin-resistance (Patti et al., 2018) and blood-pressure issues (Fogacci et al., 2019). The key problems connected to the preventive therapy using RSV are due its low oral bioavailability associated to a short serum/plasma half-life (Rege et al., 2014). However, medicinal new technologies seem to be capable to increase oral RSV bioaccessibility (Bonferoni et al., 2017; Ethemoglu et al., 2017). Although the majority of polyphenols seem to present an extremely low bioavailability after oral administration, several evidence demonstrate the beneficial effects obtained by this administration route. One of the hypothesis for the low bioavailability is the rapid metabolization of the polyphenol’s compounds, including resveratrol. However, evidence support that the resveratrol’s metabolites may also have the therapeutic properties, in addition to be found in higher concentrations in the tissues, including the central nervous system, than in the plasma (D’Archivio et al., 2010; Almeida et al., 2016). On the other hand, resveratrol's presents a high tolerability and safety profile without major pharmacological interface (cross-reaction) of this nutraceutical with orthodox known drugs. This is specific relevant, because the greatest efficient dementia medicines are generally not well tolerated, so not being prescript for the firsts disease phases (Banach et al., 2017; Fisher et al., 2017; Zanchetti et al., 2014).

## Conclusion and perspectives

The main literature data shows that Sirtuins should be considered some of the main targets on treating cognition problems and neurodegenerative diseases. Resveratrol exhibited the ability to ameliorate memory and cognition by controlling SIRT1 through AMPK and several other molecular pathways. RSV properties include antioxidative, anti-inflammatories, anti-apoptotic regulation and autophagyc properties, as well as its skills to improve cerebral blood flow and expand synaptic plasticity. In this context, Sirtuins activation and Resveratrol may be future solutions for brain diseases treatment and elderly comorbidities.

## Conflict of interest

The authors declare that they have no competing interest/disclosure(s).
